# Impact of the Momentum pilot project on male involvement in maternal health and newborn care in Kinshasa, Democratic Republic of the Congo: a quasi-experimental study

**DOI:** 10.1186/s12905-022-02032-1

**Published:** 2022-11-18

**Authors:** Anastasia J. Gage, Francine E. Wood, Madeline Woo, Rianne Gay

**Affiliations:** 1 Department of International Health and Sustainable Development, School of Public Health and Tropical Medicine, Tulane University, 1440 Canal Street, Suite 2226, New Orleans, LA 70112-2824 USA; 2 Center on Gender Equity and Health, University of California San Diego, 9500 Gilman Drive, La Jolla, CA 92093 USA; 3 Department of Health Policy and Management, School of Public Health and Tropical Medicine, Tulane University, 1440 Canal Street, New Orleans, LA 70112-2824 USA; 4 Tulane International, LLC, 14 Avenue Sergent Moke/Socimat-Concession Safricas, Kinshasa-Ngaliema, Democratic Republic of the Congo

**Keywords:** Male involvement, Maternal health, Newborn care, Gender, Antenatal care

## Abstract

**Background:**

The World Health Organization recommends that programs that seek to improve maternal and newborn health outcomes actively involve men during pregnancy, childbirth, and postpartum. However, there is little evidence on what strategies work to increase male knowledge of and involvement in antenatal and postnatal care. This study assessed the impact of the Momentum project on male involvement in maternal health and newborn care. The project involved monthly home visits to a cohort of first-time mothers aged 15–24 recruited at six-months gestation and group education sessions for their male partners using the Program P toolkit. Participants were followed-up for 16 months.

**Methods:**

The study used a quasi-experimental design with three intervention and three comparison health zones. Baseline data were collected in 2018 and endline data in 2020. Exploratory factor analysis was used to develop scales of male involvement. We measured the causal influence of Momentum using an intent-to-treat analysis at the health-zone level and a dose–response analysis at the individual level. We used random-effects probit and linear models for outcomes measured at baseline and endline, and treatment effects models with inverse-probability weighting for outcomes measured only at endline. The impact analysis involved 1,204 male partners of first-time mothers with live births.

**Results:**

Intervention health zones were associated with an 18.1 percentage point (95% CI [(10.6, 25.6]) increase in knowledge of three or more obstetric danger signs and a 13.9 percentage point (95% CI [6.3, 21.6]) increase in knowledge of newborn danger signs. Significant increases in male involvement in antenatal care (average treatment effect (ATE) = 0.728, 95% CI [0.445, 1.010]), birth planning (ATE = 0.407, 95% CI [0.157, 0.657]), and newborn care (ATE = 0.690, 95% CI [0.359, 1.021]) were found. The magnitude of Momentum’s impact increased steadily with the number of prenatal home visits and was statistically significant for all behavioral outcomes except shared decision making. Exposure to both home visits and group education sessions during the prenatal period had a significant impact on all outcomes relative to no exposure.

**Conclusions:**

The study demonstrated the effectiveness of Momentum on male involvement in maternal health and newborn care.

**Supplementary Information:**

The online version contains supplementary material available at 10.1186/s12905-022-02032-1.

## Background

Globally, there is widespread recognition of the importance of male involvement interventions as avenues for improving maternal and child health (MCH) outcomes. The 1994 International Conference on Population and Development called on countries to develop plans and strategies to engage men in reproductive health care, family planning (FP), and maternal health care [1]. More recently, the World Health Organization (WHO) [2] recommended that MCH programs actively involve men during pregnancy, childbirth, and the postpartum period “to facilitate and support improved self-care of women, improved home care practices for women and newborns, improved use of skilled care during pregnancy, childbirth and the postnatal period for women and newborns, and increase the timely use of facility care for obstetric and newborn complications” (p.3). However, little is known about what strategies are effective in promoting male involvement in maternal and newborn health (MNH) care [3, 4], especially in settings where entrenched gender norms and roles may constrain men’s participation in activities and domains that are culturally considered “female.”

Whereas studies of male involvement in low- and middle-income countries have historically focused on the prevention of mother-to-child transmission of HIV [5], a growing literature documents positive effects of male involvement on: (a) women’s access to antenatal and postnatal services [3, 6–8]; (b) adequate antenatal care (ANC) utilization [9]; (c) initiation of ANC during the first trimester [10]; (d) knowledge of danger signs [3]; (e) skilled attendance at birth and institutional delivery [3, 11]; and (f) care seeking from medically-trained providers for obstetric complications [12]. Research also suggests positive associations between men’s knowledge about maternal, infant and child feeding practices, childhood immunization, and care seeking for childhood illness. The impact of male involvement interventions on mortality, morbidity and breastfeeding was less clear [7].

Given men’s role as primary decision makers within households, families and social institutions, male involvement strategies have the potential to address gender inequities by supporting and promoting men as partners and fathers, and as change agents to challenge existing norms around decision making, masculinity, and parenthood. Strategies and interventions have typically included mass media campaigns, community-based outreach, and education for men only or couples, home visits, facility-based counseling for couples, groups, or men only, and workplace-based education [7]. To date, there has been inadequate assessment and documentation of the gender-transformative potential of involving men in reproductive, maternal, and child health [13, 14]. Only a few studies have measured the impact of male involvement interventions on women’s autonomy and empowerment, intimate partner violence, couple communication, and shared decision making [14]. There is clearly a need for rigorous evaluation to identify not only effective strategies for engaging male partners in MNH, but also potential adverse effects of male involvement programs on women’s autonomy and decision making.

Democratic Republic of the Congo (DRC), one of the largest countries in sub-Saharan Africa, has a high burden of maternal and under-five mortality, estimated at 473 per 100,000 live births in 2017 and 81 per thousand live births in 2020, respectively, according to the World Health Statistics 2022 report [15]. Early, rapid, and repeat pregnancies are common as indicated by the high adolescent birth rate (109 births per 1,000 women aged 15–19 years in the period 2012–2020 [15]), and the substantial proportion of women with short birth intervals (less than 24 months) in the DRC (27%) [16]. For half of currently married women, husbands are the sole decision makers for issues pertaining to women’s own health care [17] and almost two-thirds of men and half of women think that a man should have the final say in all family matters [18]. Traditional gender norms around pregnancy and childbearing influence male involvement in pregnancy and childbirth. Strategies to promote men’s participation in maternal, child and women’s health, including services for the prevention of mother-to-child transmission of HIV, have included community dialogue and social mobilization, creating and strengthening of male peer leaders, facility incentives such as shorter waiting time, altering clinic hours to facilitate access by men, and creating family support groups [19].

The present study contributes to filling the gap in the evidence base on effective interventions to promote male involvement in MCH. We sought to estimate the efficacy of the Momentum project, a gender-integrated FP/MNH and nutrition intervention for first-time mothers (FTMs) age 15–24 and their male partners, on male partner involvement in maternal health and newborn care. A secondary objective of our analysis was to determine if there were differences in project impact among subpopulations of younger (age 15–24) and older (age 25 and older) male partners. The paper’s contribution to the literature lies in its assessment of the extent to which the interventions were potentially gender transformative, that is, the extent to which they helped change gender relations and norms around pregnancy, childbirth, and infant care that have traditionally defined the nature and level of male involvement in maternal and infant health.

## Methods

### Study design

Momentum was a quasi-experimental community-based pilot project conducted in three intervention health zones (Kingasani, Lemba, and Matete) and three comparison health zones (Bumbu, Masina I, and Ndjili) in Kinshasa. Health zones were selected based on similar sociodemographic characteristics that could affect project outcomes (e.g., ethnicity, socioeconomic status, education, population density, access to services), the presence of a facility-based MCH project that was funded by the same donor as Momentum to facilitate referral linkages, and the absence of other projects targeting FTMs and implementing gender-transformative interventions for young parents. The project used 150 third-year nursing students (75 males and 75 females) from 11 nursing schools (*Institut Technique Médical*) to test a gender-integrated model of community-based delivery, focusing on FTMs age 15–24 and their male partners. The objectives of the project were to increase postpartum FP uptake and the adoption of MNH care seeking behaviors and household practices beneficial to mother and baby and promote more gender-equitable behaviors and attitudes among FTMs and their male partners.

### Momentum project interventions

The Momentum intervention included: (1) home visits; (2) group education sessions; and (3) social and behavioral change communication (SBCC). Home visits included not only the monitoring of healthcare seeking and health behaviors of FTMs, but also the promotion of couple communication about postpartum FP, MNH and nutrition and infant care, and shared decision making. Monthly fathers group meetings structured around Program P (an approach for engaging men in fatherhood, caregiving, and MCH) provided opportunities for reflection and dialogue during group education sessions. Program P sessions aimed at promoting male engagement in FP/MNH and nutrition and transforming gender-related attitudes, and beliefs held by male partners [20]. The sessions included the following topics: father’s expectations; father’s impact/legacy; pregnancy; birth; FP; care giving; gender; non-violence; children’s needs and rights; and dimensions of care giving. Program P sessions targeted at male partners were complemented by Program M group education sessions for FTMs that promoted awareness of human, sexual, and reproductive rights. Program M is a model for promoting young women’s awareness about gender inequities, rights, and health, and for empowering them to make decisions about different aspects of their lives [21].

The project also engaged male partners directly through video storytelling on topics related to gender norms and decision making. These videos were used to facilitate group discussions on those topics. Community dialogue sessions in the SBCC component targeted mothers, mothers-in-law, and other household influencers and aimed to influence intrahousehold dynamics in support of FTMs’ agency and equitable gender relations. Street theatre was conducted to help create an enabling environment for broad-based gender and community norm change.

The project was implemented in close collaboration with Action Santé et Développement, Johns Hopkins Center for Communication Programs (the SBCC component), the *Direction de l’Enseignement des Sciences de Santé* (Directorate of Health Sciences Education of the Ministry of Health), the *Direction de Santé des Familles et des Groupes Spécifiques* (Directorate of the Health of Families and Specific Groups), and the *Ministère de Genre, Famille et Enfant* (Ministry of Gender, Family, and Children). Project interventions were implemented for 16 months in the intervention health zones. The project trained instructors from 11 participating nursing schools, trainers from the Ministry of Health and Ministry of Gender, Family, and Children, and nursing students not only in WHO recommendations for postpartum FP, MCH and nutrition, but also in the Program M manual for FTMs and Program P manual for male partners, which had been adapted to the DRC context by the Ministry of Health, the Ministry of Gender, Family and Children, and project partners. The training focused on gender-sensitive community-based service provision that promoted egalitarian decision-making between couples, respect for women’s rights and autonomy, and the positive role that male partners could play as partners and fathers. Representatives from both Ministries as well as nursing school instructors and project partners supervised students during monthly home visits and group education sessions.

### Sample size estimation

The sample size was powered to detect a minimum absolute difference of 10–15 percentage-points change in key behavioral indicators with a 99% confidence interval (margin of error = 0.01) and 99% power, assuming an attrition rate of 25%. This minimum magnitude of change in behavioral indicators was selected for practical reasons as resources for data collection would have been insufficient to measure smaller changes with adequate precision. As the project was required to report on 15 behavioral indicators related to postpartum FP, MNH and nutrition, and gender equity outcomes among FTMs and their male partners, we selected the percent of newborns to FTMs aged 15–24 years who received a postnatal care check within two days of birth, estimated at 6.5% nationwide among women younger than age 20 in the 2013–2014 DRC DHS, as the baseline value for sample size estimation. This indicator was selected because it had the lowest prevalence compared to other indicators of interest among FTMs.

Based on these assumptions, and the likelihood of resorting to cluster sampling (with a design effect of 2.0) if social conditions in Kinshasa at the start of field implementation (which occurred in the four months preceding the December 2018 general election) did not permit a cohort follow-up study, the sample size at the project design stage was estimated at 1,213 FTMs aged 15–24 years and an equal number of male partners in the intervention health zones and 1,213 FTMs aged 15–24 years and an equal number of male partners in the comparison health zones [22]. Although we were able to implement a cohort study, we did not reduce the sample size, even though statistical formula suggested a smaller sample of FTMs and male partners [23], because we wanted to know whether the Momentum project was equally effective for FTMs age 15–19 and those age 20–24. Therefore, we had to estimate separate sample sizes for each age group to ensure that an adequate sample size was obtained, working within the confines of the resources available to our program evaluation efforts, and adding a little extra cushion for non-response. We considered that the sample size required to measure changes in indicators over time was larger than the sample size needed to measure an indicator at one point in time and that a slightly larger sample would enable us to detect changes in indicators if they occurred, even if we encountered both non-response and dropout.

### Data collection

The questionnaire used for the male partner survey reflected population and health issues relevant to Momentum’s project objectives and results framework and was shaped by both Momentum’s formative (qualitative) research conducted in 2018 and input from various stakeholders from the Ministry of Health, Ministry of Gender, Family and Children, non-governmental organizations, and international donors working on FP and MCH in the DRC. The questionnaire covered a range of topics: (a) household characteristics; (b) respondent’s background; (c) reproductive history; (d) contraception and fertility desires; (e) ANC; (f) delivery and postnatal care; (g) fertility preferences; (h) gender-relations (roles, decision making, attitudes, perceived norms, and practices related to routine childcare activities; (i) intimate partner violence perpetration; and (j) exposure to the Momentum interventions. The questionnaire was translated from English into French.

The baseline survey of male partners was conducted from September 5 to November 23, 2018, and the endline survey from May 25 to August 15, 2020. Survey instruments were pretested in Kinshasa before the start of data collection and data were collected via smartphones using the SurveyCTO mobile data collection application. Inclusion criteria for male partners’ enrollment in the study were: (1) willing and mentally competent to provide consent; (2) able to speak Lingala or French; (3) resides permanently (i.e., not visiting) in the intervention/comparison health zones; (4) husband/male partner of a recruited FTM who was approximately six-months pregnant at baseline; and (5) receipt of the pregnant FTM’s consent for her husband/male partner to be involved in the study. Written informed consent was provided by all survey participants. Interviewers gave a hard copy of the informed consent form to each participant, and then read the informed consent form out loud on the screen of the programmed smartphone. Once the male partner understood the voluntary nature of the study and agreed to participate, he signed the consent screen or checked the consent box on the interviewer’s smartphone which then unlocked the survey questionnaire.

A total of 1,766 male partners were completely interviewed in the baseline survey, of whom 1,276 were completely interviewed in the endline survey (165 traveled or moved, 137 could not be located, 47 refused to participate in the endline survey, 131 were not at home, 5 had died, 3 postponed, and 2 interviews were partly completed). A unique quick response code assigned at baseline to the couple (FTM and male partner) permitted participants’ endline data to be linked to their baseline data. After matching, a total of 1,248 male partners (600 in intervention and 648 in comparison health zones) were retained, with the remaining 518 male partners (249 in intervention and 269 in comparison health zones) considered as lost to follow-up. Overall, there was a small insignificant difference in attrition between the two arms: 29.5% in the intervention health zones and 29.3% in the comparison health zones. The analysis presented here was based on 1,204 male partners who were completely interviewed in both the baseline and endline surveys, whose data could be linked to that of the FTMs, who had live-born babies, and who had no missing data on the variables analyzed in this study. None of the socioeconomic characteristics examined in the study differed significantly between the 44 male partners with missing data/non-live births and the 1,204 male partners with non-missing data and live births (see Table S1 in Additional file 1).

### Ethical and country approval

The study was approved by the Tulane University Biomedical Institutional Review Board (2018–1028) and the University of Kinshasa School of Public Health Ethics Committee (ESP/CE/066/2018). Authorization to implement the Momentum pilot project was granted by the Secretary General of the *Ministère de la Santé Publique* (MS.1251/SG/PNSR/1358/LBE/2018) on June 11, 2018.

### Variables

#### Outcomes

1. Knowledge of three or more obstetric danger signs. This binary variable was measured in both surveys and indicated whether the male partner spontaneously reported at least three of the following danger signs during pregnancy, delivery, or soon thereafter: severe headache, fever, foul discharge, placenta does not follow the baby in 30 min, swollen feet, fits or convulsions, severe bleeding, prolonged labor of 12 h or more, and baby does not come headfirst.
2. Knowledge of three or more newborn danger signs. This binary variable was measured in both surveys and indicated whether the male partner spontaneously mentioned at least three of the following newborn danger signs: high fever, fits/convulsions/shaking of the body; yellow eyes, palms, or soles of the feet; difficult or fast breathing; difficulty feeding or sucking; feels colder than normal; redness, swelling or pus around eyes; redness, swelling puss or a bad smell around the belly button or cord.
3. Gender-equitable Men (GEM) scale. To measure this index (developed by Pulerwitz and Barker [24]), in both surveys, male partners were asked whether they totally agreed, partially agreed, or disagreed with 15 statements reflecting attitudes toward gender-equitable norms and roles in intimate relationships. Examples of statements included: “A woman’s most important role is to take care of her home and cook food for her family” and “A man should have the final word about decisions in his home.” After reverse coding responses to some of the statements to reflect gender equitable attitudes, creating binary variables for all statements to reflect total agreement with gender-equitable norms and roles in intimate relationships, performing factor analyses, and dropping items with a factor loading lower than 0.3, we constructed an 11-item predicted scale measuring gender-equitable attitudes (Cronbach’s alpha = 0.718 and Kaiser-Meyer-Olkin (KMO) measure of sampling adequacy = 0.775). Items loaded on one factor with an eigenvalue of 2.32. The proportion of the variance accounted for by the retained factor was 0.995. The GEM scale ranged from -1.858 to 2.062.
4. Male involvement in MCH: Two outcomes were constructed from the endline survey data to measure male involvement in MCH as factor loadings on 20 items revealed a two-factor solution:
  a Male involvement in ANC: This index comprised the following binary items/actions: (a) sit in the consultation room with the FTM during the checkup, (b) listen to the fetal heartbeat; (c) test for HIV or sexually-transmitted infections; (d) ask the provider if the baby is healthy; (e) ask the provider if the baby is a boy or a girl; (f) ask the provider about health problems during pregnancy; (g) ask the provider about sex during pregnancy; and (h) ask the provider about what the FTM should eat during pregnancy. The KMO coefficient was 0.930, and Cronbach’s alpha was 0.932. Factor analysis yielded a one-factor solution (eigen value = 5.063) with factor loadings greater than 0.3. The first factor accounted for more than 90% of the variance. Based on these eight items, we constructed an additive index of male partner involvement in ANC. The index ranged from 0 to 8. An additive index was chosen over the predicted scale for this and other male involvement indices to facilitate data interpretation and use by program and policy audiences working to integrate male involvement in community-based health programs in the DRC.
  b Male involvement in birth planning: Using factor analysis, we identified a six-item index from responses to the following questions: (a) finding information about the pregnancy; (b) making decisions about antenatal care; (c) making a birth plan; (d) saving money for emergencies; (e) arranging transport for delivery; and (f) deciding on skilled attendance at delivery. We originally included “arranging for a blood donor” among the items but dropped this item as its factor loading was below 0.3. Factor analysis revealed a one-factor solution with an eigenvalue of 3.189. The first factor accounted for more than 90% of the variance. The KMO coefficient for the six items was 0.872 and Cronbach’s alpha was 0.867. We created an additive index ranging from 0 to 6 to measure male involvement in birth planning.
5. Male involvement in newborn care: In the endline survey, male partners with live-born babies were asked whether they were involved “a great deal” or “a bit” (coded “1”) or “not at all” (coded “0”) in various childcare activities: changing the baby’s diapers, helping/supporting feeding; helping when the baby cries; bathing the baby; playing with the baby; looking after the baby when the mother goes out or is at work; washing the baby’s clothes; cooking or preparing food; putting the baby to sleep/bed; staying at home when the child was sick; and taking the baby to the doctor. Cronbach’s alpha for these 11 activities was 0.863. All activities had factor loadings greater than 0.3 and loaded on one factor with an eigenvalue of 4.350. This factor accounted for 95% of the variance. The KMO coefficient was 0.908. An additive scale was created from the 11 items.
6. MNH shared decision making: In both the baseline and endline surveys, the male partner was asked whether the following decisions were mainly his, the FTM’s, or someone else’s decision, or whether he and the FTM decided together: when to start seeking ANC, the number of ANC visits, where to deliver the baby, how soon to start breastfeeding, whether the FTM should practice exclusive breastfeeding, how to take care of the baby’s umbilical cord, when to seek care and treatment for danger signs of the newborn, when to seek care and treatment for newborn danger signs, where to seek care and treatment for obstetric danger signs, and how long to wait after childbirth before attempting another pregnancy. Factor analysis on the endline data (to minimize truncated decision-making experiences) yielded a one-factor solution. The first factor had an eigen value of 2.932 and accounted for 96% of the variance. The KMO coefficient was 0.821. The MNH shared decision-making index was additive, consisted of 9 items, had a Cronbach’s alpha of 0.825, and ranged from 0 to 9.

#### Intervention exposure

There were four measures of intervention exposure (that is, treatment levels), the first of which was binary and indicated that the male partner resided in the intervention health zones versus the comparison health zones at baseline. The second variable measured the type of exposure to Momentum interventions that the male partner had when the FTM was pregnant: none (reference group), home visit only, group education only, and both home visits and group education. The third variable measured the total number of home visits that the male partner received when the FTM was pregnant: none (reference group), 1–2, or 3 or more. The fourth variable measured the total number of group education sessions that the male partner attended in the prenatal and postnatal periods: None (reference group), 1–2, or three or more.

#### Control variables

Control variables were measured at baseline, with two exceptions. Age was included in the analysis as reported. A household wealth index was created from housing characteristics and household possessions using principal components analysis. The index was divided into terciles (low (reference group), medium and high). We controlled for the male partner’s number of years of schooling, marital status (never married versus ever married/formally engaged (reference group)), parents’ education (i.e., whether both parents had secondary or higher schooling; no (reference group) versus yes), and whether the male partner (a) had always lived in the locality, (b) worked in the past 12 months, (c) watched television at least once a week, (d) was a first-time father, (e) had resided with his biological father up to the age of 15 (no versus yes; data not collected in the baseline survey), and (f) reported that his biological father or father figure was very involved in raising him up until age 15 (no versus yes; data not collected in the baseline survey). We controlled for the male partner’s satisfaction with his relationship with the FTM, using the Relationship Assessment Scale [25], a seven-item additive index that ranged from 7 to 35 (alpha = 0.845; KMO = 0.875). Finally, we included a control for the male partner’s perceived power in the relationship, using the power subscale of the Gender Relations Scale [26]. The power subscale comprised seven items (e.g., my partner has more say than I do about important decisions that affect us; my partner dictates who I spend my time with, etc.). For each item, responses reflecting the most power in the relationship (after reverse coding as appropriate) were assigned a value of 1 (i.e., “totally agree”) and other responses (i.e., “partially agree” and “disagree”) were assigned 0. Thereafter, items were summed to create a composite score ranging from 0 to 7, with an alpha of 0.523.

### Statistical methods

Percentages and means were calculated to summarize the data. Chi-square tests were used to examine the significance of differences in sociodemographic characteristics between male partners in the intervention health zones and those in the comparison health zones. To determine whether there was a statistically significant difference in the mean of a given outcome variable at endline compared to baseline, we used McNemar’s Test for paired samples (i.e., repeated measures from the sample group) with binary outcomes (i.e., knowledge of obstetric and newborn dangers signs) and the Paired Samples T-test for continuous outcomes that were normally distributed and had a similar spread between the two groups (i.e., the GEM scale). The Mann–Whitney U Test was used to compare the differences between the intervention health zones and the comparison health zones in outcomes that were measured only at endline and not normally distributed (i.e., male involvement in ANC index, male involvement in birth planning index, male engagement in newborn care index, and MNH shared decision-making index). The analysis was conducted separately for adolescent/young male partners (age 15–24) and older male partners to determine if the interventions were equally beneficial (or not) in both age groups. For the Mann–Whitney U test, which was performed as a two-sided test, we reported *p*-values calculated by an exact randomization test (available when the number of observations is less than or equal to 1,000) for each age group.

To measure the causal effect of the Momentum interventions on our outcomes of interest, we first conducted an intent-to-treat analysis, whereby all male partners were analyzed according to the FTM’s health zone of residence at baseline, regardless of whether the male partner received any interventions. For panel data, knowledge of danger signs and the GEM scale, we fitted random-effects probit and linear models, respectively, and conducted pairwise comparisons of average marginal effects. Each regression model controlled for baseline values of age, household wealth, education, marital status, parents’ education, lifetime residence in the area, weekly exposure to television, employment, perceived power in the relationship, being a first-time father, and relationship satisfaction. The impact of Momentum on a given outcome was expressed as the average treatment effect (ATE), which was estimated as the difference in the predicted outcome probability or linear-form outcome prediction between the intervention and comparison health zones after the project was implemented, considering the already-existing differences (at baseline) between the intervention and comparison health zones.

For outcomes that were measured only at endline, we estimated project impact using treatment effects models with inverse-probability-weighting (IPW). We modeled our binary treatment variable, residence in intervention health zones versus comparison health zones, as a logistic function and our multivalued treatment variables (i.e., type of exposure to Momentum interventions when the FTM was pregnant, number of prenatal home visits, and number of group education sessions) as a multinomial logit function. As we did not randomly assign who would receive the Momentum interventions and who would not, treatment could be related to covariates that also affected our outcomes. Therefore, all estimates were adjusted for the following covariates: age, household wealth, education, marital status, parents’ education, lifetime residence in the area, weekly exposure to television, employment, perceived power in the relationship, being a first-time father, relationship satisfaction, co-residence with the biological father up to age 15, and high level of involvement of the biological father or father figure in raising the male partner up to age 15.

The IPW estimator is based on three assumptions: (a) conditional independence of the treatment, which means that variables that affect both treatment level and outcomes are observable; (b) overlap, which ensures that data are available on each male partner in each treatment level; and (c) independent observations, which imply that the outcome and treatment for an individual male partner has no effect on the outcome and treatment for another male partner [27]. Our data met all three assumptions. For example, visual inspection of plots of the estimated densities of the probability of getting each treatment or exposure level (see Figure S1 and S2 in Additional file 1) showed that the estimated densities had most of their respective masses in areas in which they overlapped each other, and not around 0 or 1. Therefore, there was no evidence that the overlap assumption was violated. We also conducted tests after estimation of ATEs to check whether our covariates were balanced over treatment or exposure levels and obtained a *p*-value of 0.873, which signified that our treatment model balanced the covariates. The variance inflation factor was 1.23 and suggested that multicollinearity was not of concern.

When examining the impact of the number of prenatal home visits on our outcomes of interest, we included a binary variable measuring participation in group education sessions while the FTM (i.e., the female partner) was pregnant among the covariates in the outcome model. Similarly, we included receipt of any prenatal or postnatal home visit from a Momentum nursing student among the covariates when estimating the impact of the number of group education sessions on the outcomes of interest. For the observational data (i.e., outcomes measured only at endline), the ATE measures the differences in average outcomes between male partners in the intervention health zones and male partners in the comparison health zones, after controlling for other factors. A positive ATE meant that the Momentum interventions increased the average predicted outcome while a negative ATE suggested that the Momentum intervention decreased the average predicted outcome. The impact analysis was conducted in Stata version 17 [27].

## Results

### Baseline characteristics of participants

Table 1 describes the baseline characteristics of all male partners who participated in Momentum as well as the characteristics of those lost to follow-up and those remaining in the analysis. For each group of male partners, we assessed the differences in baseline characteristics between the comparison health zones and intervention health zones to assess attrition bias.

**Table 1:** Percent distribution of male partners by loss-to-follow-up status, baseline characteristics, and study arm, Kinshasa 2020

	**Male Partners Lost to Follow-up**		**Remaining Male Partners**		**All Male Partners**	
**Baseline Characteristics**	**Comparison**	**Intervention**	**Comparison**	**Intervention**	**Comparison**	**Intervention**
**Age group**		**				**
15–24	27.6	40.4	30.4	34.2	29.6	36.0
25 +	72.4	59.6	69.7	65.8	70.5	64.0
**Years of schooling**						
Low	34.3	37.2	33.3	30.7	33.6	32.6
High	65.7	62.8	66.7	69.3	66.4	67.4
**Never married**		*		*		
No	76.9	69.2	71.3	77.0	73.0	74.7
Yes	23.1	30.8	28.7	23.0	27.0	25.3
**Household wealth**		*		***		***
Low	34.7	44.8	26.8	37.6	29.1	39.7
Medium	38.1	34.8	39.3	36.2	38.9	35.8
High	27.2	20.4	33.9	26.2	32.0	24.5
**Both parents with secondary/higher education**						
No	23.1	23.6	24.8	23.9	24.3	23.8
Yes	76.9	76.4	75.2	76.1	75.7	76.2
**Watched TV at least once a week**		*				*
No	35.8	44.4	32.8	37.1	33.7	39.2
Yes	64.2	55.6	67.2	62.9	66.3	60.8
**Worked in the past 12 months**				*		*
No	17.2	22.4	15.2	19.7	15.8	20.5
Yes	82.8	77.6	84.8	80.3	84.2	79.5
**Always lived in locality**				**		
No	72.0	60.8	64.4	62.9	66.6	62.3
Yes	28.0	39.2	35.6	37.1	33.4	37.7
**First-time father**						
No	27.2	25.6	26.3	26.7	26.6	26.4
Yes	72.8	74.4	73.7	73.3	73.4	73.6
**Relationship Assessment Scale**						
Low	48.9	56.0	47.1	50.4	47.7	52.1
High	51.2	44.0	52.9	49.6	52.3	47.9
**Gender Relations Power sub-Scale**		***				***
Low	19.8	37.6	19.4	29.5	19.5	31.9
High	80.2	62.4	80.6	70.5	80.5	68.1
Total	100.0	100.0	100.0	100.0	100.0	100.0
N	268	250	649	599	917	849

^***^ *p* < 0.001; ** *p* < 0.01; * *p* < 0.05

An examination of the baseline characteristics of the whole sample shows that, compared to male partners in the comparison health zones, more of those in the intervention health zones were adolescents/youth, residing in the poorest households, watching TV less often than once a week, unemployed, and having less gender-equitable attitudes. While the between-group differences of those lost to follow-up tended to be similar to the differences observed in the total sample, more male partners who resided in the poorest households, were never married, and had low perceived power were lost from the intervention health zones than from the comparison health zones. These differential attrition rates led to varying changes in the characteristics of those remaining in the sample. For example, in comparison health zones, more of those remaining in the sample had always lived in the locality.

When the data were disaggregated by age group, there were no statistically significant differences in baseline characteristics between those lost to follow-up and those who continued to participate in the study among male partners age 15–24. Among older male partners and in the overall sample, only one baseline characteristic, household wealth, differed significantly between those lost to follow-up and those who continued to participate in the study. More male partners who were lost to follow-up lived in the poorest households: 40% versus 32% in the overall sample (see Table S2 in Additional file 1).

As Table 1 shows, among male partners remaining in the analysis, about a third were younger than age 25 and twice as many had a high number of years of schooling and weekly TV exposure. At least one in five had never been married. Residence in the poorest households and in the locality since birth were significantly more common in the intervention health zones than in the comparison health zones (38% versus 27% and 39% versus 28%, respectively). At least four in five male partners were employed in the past 12 months, about seven in 10 were first-time fathers, and half were highly satisfied with their relationship with the FTM. Fewer male partners in intervention health zones had high perceived levels of personal agency or power in the relationship (71%) than those in the comparison health zones (81%).

### Exposure to momentum interventions

Table 2 presents data on the type and level of exposure to Momentum interventions by age group and study arm and assesses (a) whether there were male partners in comparison health zones who received Momentum interventions and (b) if so, the type of intervention received. This assessment was important as this could potentially reduce the observed impact of the project. In the comparison health zones, less than five percent of male partners were exposed to Momentum interventions, and most of them were 25 years or older. During the prenatal period, less than one percent of male partners in comparison health zones received home visits, four percent received group education sessions, and less than one percent participated in both interventions. In comparison health zones, a participation rate around three percent was observed for each group education session theme.

**Table 2:** Percent distribution of male partners by exposure to Momentum interventions, age group, and study arm, Kinshasa 2020

Type and Level of Exposure	Study Arm							
	**Comparison**				**Intervention**			
	**15–24**	**25 + **	**Total**	**N**	**15–24**	**25 + **	**Total**	**N**
**Type of exposure during the prenatal period**								
None	95.4	94.9	95.1	599	45.5	48.9	47.7	274
Home visit only	1.0	0.7	0.8	5	34.9	36.4	35.9	206
Group education only	3.1	4.4	4.0	25	5.1	2.7	3.5	20
Both	0.5	0.0	0.1	1	14.7	12.0	12.9	74
**Number of home visits in the prenatal period**								
None	98.5	99.3	99.1	624	56.1	55.6	55.8	320
1–2	1.5	0.7	1.0	6	28.3	26.9	27.4	157
3 +	0.0	0.0	0.0	0	15.7	17.6	16.9	97
**Total number of group education sessions**								
None	94.4	95.6	95.9	604	65.2	66.2	65.8	378
1–2	3.6	4.4	4.1	26	22,2	20.8	21.3	122
3 +	0.0	0.0	0.0	0	12.6	13.0	12.9	74
Total	100.0	100.0	100.0		100.0	100.0	100.0	
**Group education sessions attended (%)**								
Father’s expectations	3.1	3.0	3.0		30.3	29.3	29.6	
Father’s impact/legacy	3.1	3.2	3.2		31.3	29.3	30.0	
Pregnancy	3.1	3.2	3.2		33.8	31.4	32.2	
Birth	3.1	3.2	3.2		32.3	31.1	31.5	
Family planning	3.1	3.2	3.2		34.9	32.2	33.1	
Care giving	3.1	3.0	3.0		30.3	28.2	28.9	
Gender	2.6	3.0	2.9		22.7	24.5	23.9	
Non-violence	3.1	3.0	3.0		30.3	29.8	30.0	
Children’s needs and rights	2.6	3.2	3.0		29.8	27.4	28.2	
Dimensions of care giving	2.6	2.8	2.7		23.7	23.1	23.3	
N	195	435	630		198	376	574	

Data pertain to male partners with live-born babies

In intervention health zones, more than a third of male partners did not participate in the Momentum interventions, while 31% had partial exposure (that is, exposure to only one project component) and 30% had full exposure (that is, to both components) (data not shown). More than a quarter of male partners in the intervention health zones participated in 1–2 home visits in the prenatal period but over half did not receive a prenatal home visit. The rate of exposure to only one intervention in the prenatal period was higher for home visits (36%) than for group education sessions (four percent). Only 13% of male partners in intervention health zones received both Momentum interventions in the prenatal period. Regarding exposure to group education sessions, 30% to 33% of male partners attended most sessions, but the exposure rate was lowest for the sessions on Gender (24%) and Dimensions on Care Giving (23%).

### Bivariate analysis

Table 3 presents unadjusted mean outcomes of male involvement in MCH by age group and study arm. We used the McNemar Test to determine whether the proportion of male partners who knew three or more obstetric and three or more newborn danger signs increased significantly between the baseline and the endline surveys. The test showed that in the intervention health zones, the proportions who knew three or more obstetric danger signs were significantly different in the two surveys in both age groups (age 15–24: *p* < 0.001; age 25 and older: *p* = 0.008) and in the total sample (*p* < 0.001). In the comparison health zones, the *p*-values suggested that, within each age group, there was no difference in the proportion who knew three or more obstetric danger signs or three or more newborn danger signs between the baseline and endline surveys. However, when both age groups were combined, the *p*-value associated with the chi-square statistic was 0.027 in the comparison health zones, suggesting that the proportion who knew three or more obstetric danger signs was significantly lower at endline than at baseline (33% versus 40%). As our sample was not a random sample, these results must be interpreted with caution.

**Table 3:** Unadjusted mean male involvement outcomes, by age group and study arm, Kinshasa 2020

	**15–24**		**25 + **		**Total**	
**Outcomes**	**Comparison**	**Intervention**	**Comparison**	**Intervention**	**Comparison**	**Intervention**
**Knowledge of three or more obstetric danger signs (%)**						
Baseline	40.5	18.7	39.2	30.8	39.5	26.7
Endline	31.8	34.8	34.0	40.4	33.3	38.5
*p*-value ^a^	0.107	< 0.001	0.143	0.008	0.027	< 0.001
**Knowledge of three or more newborn danger signs (%)**						
Baseline	36.4	22.7	45.7	34.8	42.9	30.7
Endline	36.4	37.9	43.9	46.0	41.6	43.2
*p*-value ^a^	1.000	0.002	0.626	0.002	0.683	< 0.001
**Gender-equitable Men Scale (Mean)**						
Baseline	-0.117 [-0.893]	-0.240 [0.774]	0.073 [0.910]	-0.032 [0.855]	0.014 [0.908]	-0.104 [0.833]
Endline	-0.106 [0.868]	-0.101 [0.807]	0.092 [0.854]	0.118 [0.771]	0.030 [0.862]	0.043 [0.790]
*p*-value ^b^	0.900	0.038	0.729	0.005	0.723	< 0.001
**Male involvement in ANC index (Mean)**						
Endline	0.821 [1.890]	1.499 [2.453]	1.211 [2.395]	1.846 [2.784]	1.090 [2.256]	1.723 [2.678]
z, *p*-value ^c^	z = -2.950, *p* = 0.003		z = -3.293, *p* = 0.001		z = -4.308, *p* < 0.001	
**Male involvement in birth planning index (Mean)**						
Endline	2.569 [2.286]	3.000 [2.271]	3.087 [2.214]	3.380 [2.179]	2.927 [2.247]	3.249 [2.217]
z, *p*-value ^c^	z = -1.901, *p* = 0.057		z = -1.906, *p* = 0.057		z = -2.522, *p* = 0.012	
**Male engagement in newborn care index (Mean)**						
Endline	7.113 [3.238]	7.813 [2.801]	7.708 [2.948]	8.444 [2.748]	7.524 [3.050]	8.226 [2.781]
*p*-value ^c^	z = -2.008, *p* = 0.045		z = -3.996, *p* < 0.001		z = -4.245, *p* < 0.001	
**MNH shared decision-making index (Mean)**						
Endline	1.517 [2.143]	1.662 [2.175]	2.191 [2.314]	2.144 [2.311]	1.983 [2.282]	1.977 [2.275]
z, *p*-value ^c^	z = -1.674, *p* = 0.094		z = -0.238, *p* = 0.812		z = -0.851, *p* = 0.395	
N	195	198	435	376	630	574

Data pertain to male partners with live-born babies. Parenthesis = Standard deviation

^a^ *p*-values based on the McNemar’s chi-square test. Two-sided *p*-values and the exact McNemar significance probability are reported

^b^ Two-tailed *p*-value based on the Paired Samples T-test

^c^ *p*-value based on the Mann–Whitney U (Two-sample Wilcoxon Rank-sum) test. The exact *p*-value is computed for the 15–24 and 25 and older age groups, but not for the total sample as it is greater than 1,000

A Paired Samples T-test was run by study arm and age group to determine whether there was a statistically significant mean difference in the GEM score at endline compared to the baseline. Male partners in the intervention health zones had higher mean scores at endline compared to the baseline, irrespective of age group. For example, when both age groups were combined, the mean GEM score at endline was 0.043 ± 0.790 compared to -0.104 ± 0.833 at baseline, a statistically significant increase of 0.146 ((95% CI, 0.065 to 0.227), t(573) = 3.539)(not shown)), *p* < 0.001 (two-tailed)).

For outcomes that were measured only at endline and were not normally distributed, the Mann–Whitney U test was conducted to determine if the intervention led to a difference in the mean indices of male involvement. For each age group, we reported *p*-values calculated by an exact randomization two-sided test as the sample size for each age group was smaller than 1,000. Overall, the mean indices of male involvement in ANC and birth planning and the mean index of male engagement in newborn care were higher at endline than at baseline. Results showed that the mean index of male involvement in ANC was statistically significantly different between the intervention and comparison health zones (age 15–24: z = -2.950, *p* = 0.003; age 25 and older: z = -3.293, *p* = 0.001; total: z = -4.308, *p* < 0.001). Similar results were obtained for the index of male engagement in newborn care: the *p*-values of the test were smaller than our significance level of 0.05, signifying that in each age group and the total sample, the true means of the  indices were different between the study arms. Results also showed that the mean index of male involvement in birth planning and the mean index of MNH shared decision-making were not statistically significantly different between the two study arms, with one exception. The Momentum intervention had a significant impact on the mean index of male involvement in birth planning when both age groups were combined (z = -2.522, *p* = 0.012).

### Impact analysis

Table 4 presents measures of the impact of the Momentum project on male partner involvement in maternal and newborn care. As mentioned earlier, project impact was expressed as ATEs. For all outcomes, the statistical models controlled for single years of age, household wealth, male partner’s years of schooling, marital status, both parents with secondary/higher education, always lived in locality of residence, employed in the past 12 months, weekly exposure to television, first-time father, relationship satisfaction, and perceived power in the relationship. Treatment effects models for outcomes that were measured only at endline included additional controls for residence with the biological father and high father involvement while growing up.

**Table 4:** Average treatment effects (ATE) for male involvement outcomes, by age group, Kinshasa 2020

Outcome	15–24	25 +	Total
**Knowledge of 3 or more obstetric danger signs **^**a**^			
ATE	0.250	0.146	0.181
95% CI	(0.124, 0.376)	(0.054, 0.239)	(0.106, 0.256)
*P*-value	< 0.001	0.002	< 0.001
N	393	811	1,204
**Knowledge of 3 or more newborn danger signs **^**a**^			
ATE	0.150	0.131	0.139
95% CI	(0.022, 0.278)	(0.037, 0.225)	(0.063, 0.216)
*P*-value	0.022	0.007	< 0.001
N	393	811	1,204
**Gender-equitable Men’s scale **^**a**^			
ATE	0.129	0.132	0.130
95% CI	(-0.087, 0.344)	(-0.019, 0.280)	(0.007, 0.252)
*P*-value	0.243	0.087	0.038
N	393	811	1,204
**Male involvement in ANC **^**b**^			
ATE	0.838	0.733	0.728
95% CI	(0.401, 1.276)	(0.368, 1.098)	(0.445, 1.010)
*P*-value	< 0.001	< 0.001	< 0.001
N	393	811	1,204
**Male involvement in birth planning **^**b**^			
ATE	0.503	0.334	0.407
95% CI	(0.049, 0.957)	(0.034, 0.635)	(0.157, 0.657)
*P*-value	0.030	0.029	0.001
N	393	811	1,204
**Male engagement in newborn care **^**b**^			
ATE	0.716	0.708	0.690
95% CI	(0.094, 1.338)	(0.318, 1.098)	(0.359, 1.021)
*P*-value	0.024	< 0.001	< 0.001
N	393	811	1,204
**MNH shared decision-making index **^**b**^			
ATE	0.097	-0.006	0.008
95% CI	(-0.243, 0.437)	(-0.296, 0.285)	(-0.218, 0.234)
*P*-value	0.575	0.970	0.945
N	393	811	1,204

Data pertain to male partners with live-born babies

^**a**^ Based on random-effects probit and linear models with pairwise comparisons of average marginal effects. The analysis controls single years of age, household wealth, number of years of schooling, marital status, both parents with secondary/higher education, always lived in locality of residence, employed in the past 12 months, weekly exposure to television, first-time father, relationship satisfaction, and perceived power in the relationship

^**b**^ Based on treatment effects models with inverse-probability-weighting. The analysis controls for all characteristics listed in footnote ^a^ plus residence with biological father and high father involvement while growing up

The analysis showed that Momentum had a significant positive impact on six of the seven outcomes considered. The likelihood of knowing three or more obstetric danger signs and three or more newborn danger signs was higher in the intervention health zones than the comparison health zones (ATE = 0.181 and 0.139, respectively). Similarly, male partners in the intervention health zones had significantly higher gender equitable attitudes than their peers in the comparison health zones, with scores being 0.130 units higher in the intervention health zones. The ATEs for behavioral outcomes suggest that Momentum’s impact was greater for involvement in ANC compared to involvement in birth planning and engagement in newborn care. Male partners in the intervention health zones were involved in an average of 0.728 more ANC activities than those in the comparison health zones, while for birth planning and newborn care, the average number of activities were 0.407 and 0.690 units higher in the intervention health zones, respectively. No impact was observed for male partner’s shared decision making in MNH.

Except for gender-equitable attitudes and shared decision making, Momentum had a significant impact on knowledge of danger signs and involvement in maternal and newborn care among both older and younger male partners (see also impact estimates for knowledge of specific danger signs in Table S3 and Table S4 in Additional file 1). The ATE for five of our indicators was higher in the 15–24 age group, suggesting that the magnitude of the effect was greater for younger male partners. For instance, in the intervention health zones, male partners age 15–24 were involved in an average number of 0.838 more ANC activities than in the comparison health zones, while among older male partners, those in the intervention health zones were involved in an average of 0.733 more ANC activities than those in the comparison health zones.

Although Momentum’s impact on gender-equitable attitude was not observed when the data were analyzed separately by age group, further analyses revealed that the intervention had some impact on individual components of the GEM scale (see Table S5 in Additional file 1). As expected, among the younger male partners, disagreement with the statement “changing diapers, giving a bath, and feeding kids is the mother’s responsibility,” was significant higher, by 32 percentage points, in the intervention health zones than in the comparison health zones. Among older male partners, disagreement with the statements “you don’t talk about sex, you just do it” and “to be a man, you need to be tough” was significantly higher in the intervention health zones than in comparison health zones by 20 percentage points and 32 percentage points, respectively. For the ATEs corresponding to individual components of the male involvement and shared MNH decision-making indices, see Table S6 in Additional file 1.

#### Type and level of exposure

Table 5 shows the impact of the type and level of exposure to Momentum on behavioral outcomes of interest. To describe the causal impact of the level of exposure to Momentum on our outcomes, two parameters are shown: the potential-outcome mean (POM) and the ATE. The POM is the average of the predicted outcome for each level of exposure (i.e., treatment level), adjusting for covariates. The ATE is estimated by contrasting these POMs. The ATE in column (3) is the contrast in the POMs when everyone gets the specific level of treatment (exposure to Momentum) and when no one gets the treatment or is exposed to Momentum (the base level). For example, when examining male involvement in ANC, the ATE for 1–2 home prenatal home visits (0.893) is estimated as the POM for 1–2 home visits (2.123) minus the POM for no prenatal home visits/none (1.230).

**Table 5:** Type of exposure and dose–response for male involvement in maternal health and newborn care, Kinshasa 2020

**Type and Level of Exposure/Treatment**	**Potential** **Outcome Mean**	**ATE**		**ATE as Percentage Relative to the Base-level POM**		**ATE as Incremental Increase with Previous Level**	
**Male Involvement in ANC**							
Type of exposure when FTM was pregnant							
None (base level)	1.182 (1.029, 1.334)	-		-		-	
Home visit only	2.106 (1.709, 2.504)	0.925 (0.500, 1.350)	***	78.2%	***	0.925 (0.500, 1.350)	***
Group education only	1.267 (0.550, 1.985)	0.085 (-0.646, 0.817)		7.2%		-0.839 (-1.656, -.023)	*
Both home visit and group education	2.254 (1.621, 2.888)	1.073 (0.421, 1.724)	***	90.8%	***	0.987 (0.032, 1.943)	*
Number of prenatal home visits ^a^							
None	1.230 (1.077, 1.384)	-		-		-	
1–2	2.123 (1.638, 2.609)	0.893 (0.386, 1.400)	***	7260.0%	***	0.893 (0.386, 1.400)	***
3 +	2.315 (1.677, 2.953)	1.085 (0.429, 1.740)	***	88.2%	***	0.192 (-0.610, 0.993)	
**Male involvement in birth planning**							
Type of exposure when FTM was pregnant							
None (base level)	2.748 (2.601, 2.894)	-		-		-	
Home visit only	3.796 (3.520, 4.072)	1.048 (0.737, 1.359)	***	38.1%	***	1.048 (0.737, 1.359)	***
Group education only	4.022 (3.515, 4.528)	1.279 (0.747, 1.801)	***	46.4%	***	0.226 (-0.350, 0.802)	
Both home visit and group education	4.255 (3.851, 4.659)	1.507 (1.078, 1.936)	***	54.9%	***	0.233 (-0.413, 0.879)	
Number of prenatal home visits ^a^							
None (base level)	2.931 (2.786, 3.076)	-		-		-	
1–2	3.578 (3.252, 3.905)	0.647 (0.293, 1.002)	***	22.1%	***	0.647 (0.293, 1.002)	***
3 +	4.300 (3.844, 4.756)	1.369 (0.896, 1.843)	***	46.7%	***	0.722 (0.163, 1.280)	**
**Male engagement in newborn care**							
Type of exposure when FTM was pregnant							
None (base level)	7.611 (7.409, 7.814)	-		-		-	
Home visit only	8.697 (8.365, 9.030)	1.086 (0.670, 1.473)	***	14.3%	***	1.086 (0.670, 1.473)	***
Group education only	8.315 (7.652, 8.979)	0.704 (0.010, 1.398)	*	9.2%	*	-0.382 (-1.124, 0.360)	
Both home visit and group education	8.479 (7.960, 8.998)	0.867 (0.311, 1.423)	**	11.4%	*	0.164 (-0.678, 1.005)	
Number of prenatal home visits ^a^							
None (base level)	7.683 (7.491, 7.875)	-		-		-	
1–2	8.422 (7.975, 8.868)	0.739 (0.254, 1.224)	**	9.6%	**	0.739 (0.254, 1.224)	**
3 +	8.973 (8.558, 9.387)	1.290 (0.834, 1.745)	***	16.8%	***	0.551 (-0.057, 1.159)	
Total number of group education sessions (prenatal and postnatal) ^b^							
None (base level)	7.816 (7.633, 7.999)	-		-		-	
1–2	7.671 (7.065, 8.277)	-0.145 (-0.777, 0.487)		-1.9%		-0.145 (-0.777, 0.487)	
3 +	8.912 (8.222, 9.601)	1.096 (0.383, 1.809)	**	14.0%	**	1.241 (0.326, 2.155)	**
**Shared MNH decision making**							
Type of exposure when FTM was pregnant							
None (base level)	1.400 (1.265, 1.535)	-		-		-	
Home visit only	1.607 (1.336, 1.878)	0.207 (-0.087, 0.501)		14.8%		0.207 (-0.087, 0.501)	
Group education only	3.115 (2.243, 3.986)	1.715 (0.836, 2.593)	***	122.5%	***	1.508 (0.597, 2.418)	***
Both home visit and group education	1.963 (1.515, 2.412)	0.564 (0.101, 1.026)	*	40.3%	*	-1.151 (-2.129, -0.174)	*
Number of prenatal home visits ^a^							
None (base level)	1.558 (1.412, 1.704)	-		-		-	
1–2	1.638 (1.311, 1.964)	0.080 (-0.268, 0.427)		5.1%		0.080 (-0.268, 0.427)	
3 +	1.809 (1.296, 2.323)	0.251 (-0.273, 0.775)		16.1%		0.172 (-0.432, 0.775)	
Total number of group education sessions (prenatal and postnatal) ^b^							
None (base level)	1.441 (1.307, 1.576)	-		-		-	
1–2	2.662 (2.060, 3.263)	1.221 (0.613, 1.828)	***	84.7%	***	1.221 (0.613, 1.828)	***
3 +	2.035 (1.159, 2.910)	0.593 (0.291, 1.478)		41.2%		-0.627 (-1.683, 0.429)	

Data pertain to male partners with live-born babies. The analysis controls for the following characteristics: single years of age, household wealth, male partner’s years of schooling, marital status, both parents with secondary/higher education, always lived in locality of residence, employed in the past 12 months, weekly exposure to television, first-time father, relationship satisfaction, perceived power in the relationship, residence with biological father while growing up, and high father involvement while growing up. *N* = 1,203 for all models. Parenthesis = 95% CI. Based on multivalued treatment effects models with inverse-probability-weighting

^a^ Controls for participation in Momentum group education (Program P) sessions while the FTM (i.e., the female partner) was pregnant. Data were not collected on the number of group education sessions the male partner attended while the FTM was pregnant, but on the total number of sessions attended

^b^ Controls for the exposure to any home visits during the life of the project

^***^ *p* < 0.001; ** *p* < 0.01; * *p* < 0.05

To aid interpretation, we expressed the ATE as a percentage of the POM obtained for those who did not receive the intervention (i.e., the untreated or base level). We contrasted the POM for each level of exposure with the POM for the previous level—reverse-adjacent contrasts—to see if there were significant incremental increases in the ATE as the number of prenatal home visits and number of group education sessions increased. We used a similar approach to see if there were significant differences in the ATE between (a) exposure to group education only and exposure to home visits only; and (b) exposure to both home visits and group education and exposure to group education only.

Regarding the type of exposure to Momentum in the prenatal period, participation in both home visits and group education sessions (that is, full exposure) had a significant impact on all outcomes relative to no exposure. The estimated ATE of going from no exposure to full exposure was 1.073 (95% CI: 0.421, 1.724) for involvement in ANC; 1.507 (95% CI: 1.078, 1.936) for involvement in birth planning; 0.867 (95% CI: 0.311, 1.423) for engagement in newborn care; and 0.564 (95% CI: 0.101, 1.026) for shared MNH decision making. The ATE for exposure to home visits alone relative to no exposure was statistically significant for involvement in ANC, involvement in birth planning, and engagement in newborn care. Exposure to home visits alone had no impact on shared decision making while exposure to group education sessions alone when the FTM was pregnant had no impact on involvement in ANC. Exposure to prenatal group education sessions alone (relative to no exposure) had a significant impact on involvement in birth planning, engagement in newborn care, and shared MNH decision making. Expressing the ATE as a percentage of the base-level POM (“None”) indicated that the shared decision-making index was 123% higher (estimated as (3.115 divided by 1.400) minus 1) if all male partners were exposed to group education sessions only than if no male partner was exposed to Momentum.

The ATE increased steadily with the number of prenatal home visits for all outcomes except shared decision making. An examination of the ATEs as incremental increases from the previous level (i.e., the reverse adjacent contrasts shown in column (5) of Table 5) indicated that there was no significant gain to participation in three or more home visits versus 1–2 home visits, the only exception being for involvement in birth planning, where the ATE was 0.722 (95% CI: 0.163, 1.280; *p* < 0.01).

No dose–response was detected for the impact of the number of group education sessions on the index of male engagement in newborn care and the index of shared decision making. For engagement in newborn care, the ATE suggested that the average male partner attending three or more group education sessions would have an index that was 1.096 points higher than he would have had if he had attended no group education sessions. A comparison of the ATE for three or more group education sessions with the ATE for 1–2 group education sessions suggested that, if all male partners who attended 1–2 sessions had participated in three or more sessions, the index of male engagement in newborn care would have been significantly higher (ATE = 1.241; 95% CI: 0.326, 2.144) (see the last column of Table 5). The ATE for shared decision making was 85% higher among male partners who participated in 1–2 relative to no group education sessions, but only 41% higher among those who participated in three or more group education sessions relative to none.

## Discussion

Engaging male partners in maternal health and newborn care is an important step in improving MCH outcomes. However, little is known about what strategies are effective in promoting male involvement in MCH within a package of interventions to improve care seeking, practices in the home, and postpartum FP uptake. Our analysis showed that, in intervention health zones, 52% of male partners were reached by Momentum interventions during the prenatal period and a third (34%) participated in Momentum’s group education sessions. Using a robust research design and statistical techniques that reliably enabled us to infer plausibility, this study found that the Momentum project caused a significant increase in knowledge of obstetric and newborn danger signs and gender-equitable attitudes over time and small but significant increases in involvement in ANC, and engagement in infant care among male partners of adolescent and young FTMs. Momentum’s impact on involvement in birth planning and shared decision making about MNH issues was negligible.

The evidence showed that the magnitude of the impact depended on the type and level of program exposure and the behavioral outcome examined. Exposure to both home visits and group education sessions had a significant impact on all outcomes relative to no exposure. Exposure to home visits only relative to no exposure to Momentum led to a significant improvement in all behavioral outcomes except shared decision making. Relative to no exposure to Momentum, the impact of exposure to group education only was statistically significant for all behavioral outcomes except male involvement in ANC. The magnitude of the impact increased steadily with the number of prenatal home visits and was statistically significant for all behavioral outcomes except shared decision making. The evidence showed that the average male partner attending three or more group education sessions would have an index of engagement in newborn care that was significantly higher than he would have had if he had attended no group education sessions.

Studies in sub-Saharan African and other developing countries have found that interventions aimed at increasing male involvement are effective; however, the indicators measuring male involvement have varied [28, 29]. For instance, in Tanzania, a study using home visits by community health workers to improve male involvement in maternal health had similar findings, although the measures were slightly different [30]. Like our study, this study showed significant improvement in the composite score measuring male involvement in ANC and delivery (net intervention effect (NIE) = 41%), involvement in three or more birth planning and complication readiness actions (NIE = 27%), ANC attendance (NIE = 16%), attendance at delivery (NIE = 33%), and knowledge of three or more danger signs of pregnancy, childbirth and postpartum (NIE = 27%). Our study did not show improvement in shared decision making; however, in the Tanzanian study, couple’s shared decision making increased with an NIE of 39% [30].

Although the afore-mentioned studies showed impact, it is important to note that the methods measuring impact varied as did research designs used, which could contribute to the differences in the studies’ results. A recent meta-analysis of male participation in birth preparedness and complication readiness found that in sub-Saharan Africa, 40% of male partners were involved in birth planning, with a range of 6% to 86% [31]. Another meta-analysis on the effectiveness of responsible fatherhood programs found that programs produced small but significant effects in father involvement which included any interaction the father had with his child [32].

Our dose–response analysis results are comparable to those of other studies. Authors of a systematic review of the effectiveness of interventions involving men living with HIV-positive pregnant women in low-income countries found that interventions with multiple strategies were more likely to be effective in promoting male involvement than single interventions [29]. The authors also suggested that home visit strategies were most effective for single interventions. In our study, prenatal exposure to home visits alone had a greater impact than prenatal exposure to group education sessions alone when examining male involvement in ANC and male engagement in newborn care. The reverse was the case when examining shared MNH decision making.

### Research gaps addressed

This study contributes to the literature on how male involvement can be effectively promoted in settings where socially constructed gender roles, norms and relations could effectively constrain men’s participation in MNH. We expand the literature on male involvement in low-income countries beyond a focus on the prevention of mother-to-child-transmission of HIV, to include maternal health and newborn care. The present study also sheds light on the effect of male involvement in MNH on egalitarian decision making, which has been a gap in the existing literature. Finally, our study contributes to the evaluation rigor of male involvement strategies in MCH.

### Limitations

This study has a few limitations that should be taken into consideration when reviewing the results. First, as previously mentioned, the Momentum study oversampled by 25% to account for attrition. The attrition rate for male partners in our study was 29%; however, this fell within the range of attrition rates for other fatherhood programs (30% or higher) [33–35]. Unfortunately, the endline study did not collect data on reasons for non-participation in home visits and group education sessions. This information could have helped inform strategies for increasing participation and retention of male partners of FTMs in future male involvement programs.

The second limitation of the study is the possibility of social desirability bias among respondents. Male partners in both intervention and comparison health zones may have been motivated to over report engagement in desirable behaviors, such as routine childcare or shared decision making. As project activities included the promotion of male involvement in MNH, men in the intervention health zones may have felt more pressure to give “desirable” responses, and bias may have been stronger within this group.

The third limitation is that our analysis of the impact of male involvement did not integrate any qualitative data on male partners’ or FTMs’ preferences for male engagement. These preferences could impact male participation independent of the intervention and, thus, should ideally have been controlled for. Any potential bias would likely have been non-differential as there was no reason to think that there could have been a systematic difference in preferences between the intervention and comparison health zones. Consequently, the impact of participants’ personal preferences should have made a null result more likely. We plan on presenting the findings from our qualitative research in future publications.

Fourth, treatment assignment was not randomized. FTMs were recruited at six months gestation at health facilities and in the community, and their male partners were identified through them. Although health zones were matched on sociodemographic factors that could affect project outcomes as well as the absence of other projects in the health zone targeting the same demographic, it is possible that not all confounders were controlled for in the study design. While randomization can eliminate uncontrolled confounding, this was not realistically feasible in the study setting.

Finally, the internal validity of our results could be threatened not only by omitted variable bias, but also by regression to the mean, an issue that could have affected outcomes that were of extremely low or high prevalence at baseline. There was also evidence of the effect of social interaction – 26 out of 630 male partners in the comparison health zones participated in the group education sessions. It must also be noted that the data analyzed in this study were not representative of all male partners of 15–24-year-old FTMs in Kinshasa.

## Conclusions and program recommendations

Several program recommendations stem from our results. First additional strategies must be identified for reaching and retaining male partners of adolescent and young FTMs in gender-integrated FP and MNH programs. Only half of male partners participated in prenatal home visits and a third participated in group education sessions. While the Momentum interventions were implemented by nursing students at the community level, greater male participation rates might be attained in the future if partnerships are built with schools, workplaces, and churches, and if the project’s interventions are integrated in sports clubs, health facilities, barber shops, and community-based associations. Male partner participation rates could also improve if the schedules for monthly home visits and group education sessions are adjusted to account for male partners’ work schedules and availability. In addition, male partners who participated in Momentum could be engaged in future project activities as peer leaders and agents of change to provide testimonials and encourage other expectant fathers to be meaningfully engaged in MNH.

As the prenatal period provides a window of opportunity to engage and empower expectant fathers, health facilities should complement the community-based male-involvement activities implemented by nursing students by (a) providing personalized invitations to male partners of FTMs; (b) holding open days for expectant fathers, and (c) disseminating information on pregnancy, childbirth, less known obstetric danger signs (e.g., prolonged labor lasting 12 h or more and placenta does not follow baby in 30 min, see Table S2 in Additional file 1), less known newborn danger signs (e.g., yellow eyes/palms/soles of feet; red swelling or pus around the eyes; and redness, swelling, pus, bad smell around the belly button or cord, see Table S3 in Additional file 1), the benefits of home visits, and group education topics to male audiences. Although Momentum established referral systems with clinics utilized by FTMs, there is a need to set up more effective mechanisms of collaboration and synergy between nursing schools, clinic-based providers, and community actors including the *Recos* (i.e., community mobilizers) for continuous and targeted joint actions aimed at raising awareness among male partners and encouraging them to actively participate in MNH interventions.

Our analysis detected no impact of participation in home visits alone on shared decision making about MNH issues. Furthermore, project impact on male involvement in birth planning was negligible. These results highlight the need to reinforce messages about shared decision-making during home visits and group education sessions, train both male partners and FTMs to engage in frank communication about male engagement in MNH and strengthen couples’ problem-solving skills. The lack of project impact on gender-equitable attitudes among adolescent and young male partners calls for further research to identify and understand the sources of influence and information that shape young men’s gender-related attitudes and their relationships with their female partners. While the project explored the role of key household influencers and community members in this regard, institutions such as schools and workplaces, and media channels such as radio and television were not examined during formative research. To promote enduring changes in gender-related attitudes, it would be important to garner community support to develop appropriate messages and invest in long-term community-led activities to create an enabling environment for gender-equitable attitudinal and social norm change.

The Government of the DRC has shown strong support for institutionalizing the Momentum nursing student model, which will include both classroom instruction and a field-level practicum consisting of home-based counseling, group education, and community-based service provision. It will be important to adapt the male involvement and other gender-related components of the Momentum model so that they are more cost effective, develop partnerships with other organizations working on MNH in the DRC, and mobilize resources for the male involvement and gender-related components of the adapted model. It will also be important to conduct a process evaluation of the institutionalization of the male involvement and gender-related components of the adapted Momentum model to document lessons learned and make necessary adjustments.

## Supplementary Information

**Additional file 1.**

## Data Availability

The datasets used and/or analyzed during the current study are available from the corresponding author on reasonable request.

## Abbreviations

ANC: Antenatal care
ATE: Average treatment effect
CI: Confidence interval
DRC: Democratic Republic of the Congo
FP: Family planning
FTM: First-time mother
KMO: Kaiser-Myer Olkin
MCH: Maternal and child health
MNH: Maternal and newborn health
NIE: Net intervention effect
POM: Potential outcome mean
SBCC: Social and behavior change communication
WHO: World Health Organization
