# Supplementary material for: Impact of the Momentum pilot project on male involvement in maternal health and newborn care in Kinshasa, Democratic Republic of the Congo: a quasi-experimental study
Source: BMC Womens Health. 2022 Nov 18;22:460. doi: 10.1186/s12905-022-02032-1 (PMC9673298; doi:10.1186/s12905-022-02032-1)
Supplement: Supplementary file 1 — Additional file 1. [file 12905_2022_2032_MOESM1_ESM.docx]

**SUPPLEMENTAL FIGURES**

Figure S1. Plot of the test of the overlap assumption

Figure S2. Plot of the test of the overlap assumption for prenatal exposure levels

**SUPPLEMENTAL TABLES**

**Table S1**. Mean number of years of schooling and percentage distribution of male partners who were interviewed in both the baseline and endline surveys and whose data could be linked to the data for the first-time mother, by background characteristics and non-missingness of data on variables of interest, Kinshasa 2020

| **Baseline Characteristics** | **Missing Data/Non-live Birth** | **No Missing Data and Live Birth** |
| --- | --- | --- |
| Mean number of years of schooling (SD) | 11.8 (2.932) | 11.5 (4.158) |
| Health zone |  |  |
| Control | 40.9 | 52.4 |
| Intervention | 59.1 | 47.7 |
| Never married |  |  |
| No | 75.0 | 74.0 |
| Yes | 25.0 | 26.0 |
| Household wealth |  |  |
| Low | 40.9 | 31.6 |
| Medium | 31.8 | 38.0 |
| High | 27.3 | 30.3 |
| Both parents with secondary/higher education |  |  |
| No | 25.0 | 24.3 |
| Yes | 75.0 | 75.7 |
| Watched TV at least once a week |  |  |
| No | 34.1 | 34,9 |
| Yes | 65.9 | 65.1 |
| Worked in the past 12 months |  |  |
| No | 11.4 | 17.6 |
| Yes | 88.6 | 82.4 |
| Always lived in locality |  |  |
| No | 61.4 | 63.8 |
| Yes | 38.6 | 36.2 |
| First-time father |  |  |
| No | 34.1 | 26.2 |
| Yes | 65.9 | 73.8 |
| Relationship Assessment Scale |  |  |
| Low | 43.2 | 48.9 |
| High | 56.8 | 51.1 |
| Gender relations Power sub-scale |  |  |
| Low | 20.4 | 24.4 |
| High | 79.9 | 75.6 |
| Age group |  |  |
| 15-24 | 20.4 | 32.6 |
| 25 and older | 79.6 | 67.4 |
|  |  |  |
| Total | 100.0 | 100.0 |
| N | 44 | 1,204 |

SD Standard deviation

**Table S2**. Mean number of years of schooling and percentage distribution of male partners, by background characteristics, lost-to-follow-up status, and age group, Kinshasa 2020

|  | **15-24** | |  | **25 and Older** | |  | **Total** | | |
| --- | --- | --- | --- | --- | --- | --- | --- | --- | --- |
| **Baseline Characteristics** | **LTFU** | **Not LTFU** |  | **LTFU** | **Not LTFU** |  | **LTFU** | **Not LTFU** | **N** |
| Mean number of years of schooling (SD) | 10.8  (2.766) | 10.9  (2.518) |  | 11.7  (2.970) | 11.9  (3.014) |  | 11.4  (2.929) | 11.6  (2.897) |  |
| Health zone |  |  |  |  |  |  |  |  |  |
| Control | 42.3 | 49.0 |  | 56.6 | 53.4 |  | 51.7 | 52.0 | 917 |
| Intervention | 57.7 | 51.0 |  | 43.4 | 46.6 |  | 48.3 | 48.0 | 849 |
| Never married |  |  |  |  |  |  |  |  |  |
| No | 69.1 | 72.9 |  | 75.2 | 74.6 |  | 73.2 | 74.0 | 1,303 |
| Yes | 30.9 | 27.1 |  | 24.8 | 25.4 |  | 26.8 | 26.0 | 463 |
| Household wealth |  |  |  | *** | |  | ** | |  |
| Low | 43.4 | 40.1 |  | 37.6 | 28.1 |  | 39.6 | 32.0 | 604 |
| Medium | 32.0 | 35.8 |  | 38.8 | 38.8 |  | 36.5 | 37.8 | 661 |
| High | 24.6 | 24.1 |  | 23.6 | 33.1 |  | 23.9 | 30.2 | 501 |
| Both parents with secondary/higher education |  |  |  |  |  |  |  |  |  |
| No | 18.3 | 20.1 |  | 26.0 | 26.4 |  | 23.4 | 24.4 | 425 |
| Yes | 81.7 | 79.9 |  | 74.1 | 73.6 |  | 76.6 | 75.6 | 1,341 |
| Watched TV at least once a week |  |  |  |  |  |  |  |  |  |
| No | 44.0 | 37.3 |  | 37.9 | 33.7 |  | 40.0 | 34.9 | 642 |
| Yes | 56.0 | 62.7 |  | 62.1 | 66.3 |  | 60.0 | 65.1 | 1,124 |
| Worked in the past 12 months |  |  |  |  |  |  |  |  |  |
| No | 25.1 | 26.6 |  | 16.9 | 13.0 |  | 19.7 | 17.4 | 319 |
| Yes | 74.9 | 73.4 |  | 83.1 | 87.1 |  | 80.3 | 82.6 | 1,447 |
| Always lived in locality |  |  |  |  |  |  |  |  |  |
| No | 58.9 | 58.2 |  | 70.6 | 66.3 |  | 66.6 | 63.7 | 1,140 |
| Yes | 41.1 | 41.8 |  | 29.4 | 33.7 |  | 33.4 | 36.3 | 626 |
| First-time father |  |  |  |  |  |  |  |  |  |
| No | 9.1 | 11.4 |  | 35.3 | 33.7 |  | 26.4 | 26.5 | 468 |
| Yes | 90.9 | 88.6 |  | 64.7 | 66.3 |  | 73.6 | 73.5 | 1,298 |
| Relationship Assessment Scale |  |  |  |  |  |  |  |  |  |
| Low | 61.1 | 53.2 |  | 47.8 | 46.6 |  | 52.3 | 48.7 | 879 |
| High | 38.9 | 46.8 |  | 52.2 | 53.4 |  | 47.7 | 51.3 | 887 |
| Gender relations Power sub-scale |  |  |  |  |  |  |  |  |  |
| Low | 85.7 | 81.8 |  | 81.1 | 79.1 |  | 82.6 | 80.0 | 1,426 |
| High | 14.3 | 18.2 |  | 18.9 | 20.9 |  | 17.4 | 20.0 | 340 |
|  |  |  |  |  |  |  |  |  |  |
| Total | 100.0 | 100.0 |  | 100.0 | 100.0 |  | 100.0 | 100.0 |  |
|  |  |  |  |  |  |  |  |  |  |
| N | 175 | 402 |  | 343 | 846 |  | 518 | 1,248 | 1,766 |

*** p < 0.001; ** p < 0.01 based on Pearson’s chi-square test

SD Standard deviation

LTFU Lost to follow-up

Table S3. Impact estimate for knowledge of specific danger signs during pregnancy, delivery or soon thereafter, male partners of first-time mothers, by age group, Kinshasa 2020

|  | **Adjusted Means** | | | |  | **Impact** | | |  |  |
| --- | --- | --- | --- | --- | --- | --- | --- | --- | --- | --- |
|  | **Before/Baseline** | | **After/Endline** | |  |  |  |  |  |  |
| **Component** | **Comparison** | **Intervention** | **Comparison** | **Intervention** |  | **ATE** | **95% CI** | **p-value** |  | **N** |
| Severe headache |  |  |  |  |  |  |  |  |  |  |
| 15-24 | 0.497 | 0.521 | 0.410 | 0.536 |  | 0.102 | (0.036, 0.240) | 0.149 |  | 393 |
| 25+ | 0.487 | 0.657 | 0.480 | 0.479 |  | -0.171 | (0.265, -0.076) | <0.001 |  | 811 |
| Total | 0.489 | 0.611 | 0.457 | 0.499 |  | -0.080 | (0.159, -0.001) | 0.047 |  | 1,204 |
| Fever |  |  |  |  |  |  |  |  |  |  |
| 15-24 | 0.668 | 0.650 | 0.699 | 0.706 |  | 0.024 | (0.102, 0.150) | 0.706 |  | 393 |
| 25+ | 0.738 | 0.688 | 0.733 | 0.747 |  | 0.063 | (0.023, 0.150) | 0.150 |  | 811 |
| Total | 0.716 | 0.676 | 0.722 | 0.734 |  | 0.051 | (0.021, 0.123) | 0.162 |  | 1,204 |
| Foul discharge |  |  |  |  |  |  |  |  |  |  |
| 15-24 | 0.171 | 0.073 | 0.121 | 0.129 |  | 0.107 | (0.020, 0.194) | 0.016 |  | 393 |
| 25+ | 0.217 | 0.104 | 0.117 | 0.172 |  | 0.168 | (0.101, 0.235) | <0.001 |  | 811 |
| Total | 0.203 | 0.093 | 0.118 | 0.156 |  | 0.148 | (0.095, 0.202) | <0.001 |  | 1,204 |
| Placenta does not follow  baby in 30 minutes |  |  |  |  |  |  |  |  |  |  |
| 15-24 | 0.042 | 0.010 | 0.024 | 0.016 |  | 0.024 | (0.014, 0.063) | 0.217 |  | 393 |
| 25+ | 0.069 | 0.019 | 0.053 | 0.040 |  | 0.037 | (0.023, 0.098) | 0.223 |  | 811 |
| Total | 0.061 | 0.016 | 0.045 | 0.032 |  | 0.033 | (0.003, 0.063) | 0.029 |  | 1,204 |
| Swollen feet |  |  |  |  |  |  |  |  |  |  |
| 15-24 | 0.272 | 0.139 | 0.242 | 0.222 |  | 0.113 | (0.001, 0.226) | 0.052 |  | 393 |
| 25+ | 0.230 | 0.155 | 0.225 | 0.242 |  | 0.092 | (0.015, 0.169) | 0.019 |  | 811 |
| Total | 0.244 | 0.149 | 0.232 | 0.234 |  | 0.098 | (0.034, 0.161) | 0.003 |  | 1,204 |
| Fits/convulsions |  |  |  |  |  |  |  |  |  |  |
| 15-24 | 0.125 | 0.085 | 0.036 | 0.080 |  | 0.083 | (0.008, 0.158) | 0.030 |  | 393 |
| 25+ | 0.110 | 0.098 | 0.083 | 0.080 |  | 0.008 | (0.048, 0.064) | 0.772 |  | 811 |
| Total | 0.114 | 0.094 | 0.068 | 0.080 |  | 0.032 | (0.013, 0.077) | 0.166 |  | 1,204 |
| Severe bleeding |  |  |  |  |  |  |  |  |  |  |
| 15-24 | 0.410 | 0.158 | 0.405 | 0.420 |  | 0.266 | (0.141, 0.392) | <0.001 |  | 393 |
| 25+ | 0.416 | 0.260 | 0.407 | 0.479 |  | 0.228 | (0.137, 0.319) | <0.001 |  | 811 |
| Total | 0.412 | 0.226 | 0.404 | 0.461 |  | 0.243 | (0.169, 0.316) | <0.001 |  | 1,204 |
| Prolonged labor lasting  12 hours or more |  |  |  |  |  |  |  |  |  |  |
| 15-24 | 0.015 | 0.026 | 0.059 | 0.074 |  | 0.003 | (0.050, 0.056) | 0.908 |  | 393 |
| 25+ | 0.035 | 0.055 | 0.073 | 0.058 |  | -0.035 | (0.079, 0.009) | 0.117 |  | 811 |
| Total | 0.029 | 0.045 | 0.069 | 0.063 |  | -0.022 | (0.056, 0.012) | 0.205 |  | 1,204 |
| Baby does not come out  head first |  |  |  |  |  |  |  |  |  |  |
| 15-24 | 0.086 | 0.021 | 0.035 | 0.046 |  | 0.076 | (0.019, 0.133) | 0.009 |  | 393 |
| 25+ | 0.076 | 0.044 | 0.051 | 0.050 |  | 0.030 | (0.014, 0.074) | 0.180 |  | 811 |
| Total | 0.078 | 0.036 | 0.046 | 0.049 |  | 0.045 | (0.010, 0.080) | 0.012 |  | 1,204 |

Notes: Data pertain to male partners with live-born babies. The analysis is based on panel data and is conducted with xtlogit and margins commands in Stata 17. Each model controls for the following baseline characteristics: single years of age, household wealth, male partner’s years of schooling, marital status, both parents with secondary/higher education, always lived in locality of residence, employed in the past 12 months, weekly exposure to television, first-time father, relationship satisfaction, and perceived power in the relationship.

ATE Average treatment effect

CI Confidence Interval

Table S4. Impact estimate for knowledge of specific newborn danger signs, male partners of first-time mothers, by age group, Kinshasa 2020

|  | **Adjusted Means** | | | |  | **Impact** | | |  |  |
| --- | --- | --- | --- | --- | --- | --- | --- | --- | --- | --- |
|  | **Before/Baseline** | | **After/Endline** | |  |  |  |  |  |  |
| **Component** | **Comparison** | **Intervention** | **Comparison** | **Intervention** |  | **ATE** | **95% CI** | **p-value** |  | **N** |
| High fever |  |  |  |  |  |  |  |  |  |  |
| 15-24 | 0.868 | 0.788 | 0.910 | 0.907 |  | 0.076 | (-0.017, 0.169) | 0.110 |  | 393 |
| 25+ | 0.900 | 0.827 | 0.930 | 0.932 |  | 0.075 | (0.017, 0.134) | 0.012 |  | 811 |
| Total | 0.889 | 0.816 | 0.923 | 0.924 |  | 0.075 | (0.026, 0.125) | 0.003 |  | 1,204 |
| Fits, shaking of the body |  |  |  |  |  |  |  |  |  |  |
| 15-24 | 0.339 | 0.148 | 0.181 | 0.241 |  | 0.250 | (0.136, 0.363) | <0.001 |  | 393 |
| 25+ | 0.347 | 0.199 | 0.212 | 0.247 |  | 0.183 | (0.100, 0.267) | <0.001 |  | 811 |
| Total | 0.347 | 0.180 | 0.204 | 0.243 |  | 0.205 | (0.138, 0.272) | <0.001 |  | 1,204 |
| Yellow eyes/palms/soles of feet |  |  |  |  |  |  |  |  |  |  |
| 15-24 | 0.134 | 0.115 | 0.114 | 0.161 |  | 0.067 | (-0.023, 0.156) | 0.145 |  | 393 |
| 25+ | 0.134 | 0.130 | 0.180 | 0.109 |  | -0.068 | (-0.134, -0.001) | 0.047 |  | 811 |
| Total | 0.134 | 0.125 | 0.159 | 0.127 |  | -0.024 | (-0.078, 0.030) | 0.390 |  | 1,204 |
| Difficult or fast breathing |  |  |  |  |  |  |  |  |  |  |
| 15-24 | 0.270 | 0.269 | 0.260 | 0.249 |  | -0.010 | (-0.127, 0.107) | 0.864 |  | 393 |
| 25+ | 0.362 | 0.270 | 0.307 | 0.350 |  | 0.135 | (0.047, 0.224) | 0.003 |  | 811 |
| Total | 0.331 | 0.271 | 0.291 | 0.317 |  | 0.087 | (0.016, 0.158) | 0.017 |  | 1,204 |
| Difficulty feeding or sucking |  |  |  |  |  |  |  |  |  |  |
| 15-24 | 0.300 | 0.194 | 0.371 | 0.291 |  | 0.025 | (-0.098, 0.148) | 0.688 |  | 393 |
| 25+ | 0.348 | 0.327 | 0.352 | 0.412 |  | 0.081 | (-0.010, 0.172) | 0.080 |  | 811 |
| Total | 0.334 | 0.281 | 0.358 | 0.371 |  | 0.064 | (-0.009, 0.137) | 0.085 |  | 1,204 |
| Feels colder than normal |  |  |  |  |  |  |  |  |  |  |
| 15-24 | 0.068 | 0.048 | 0.063 | 0.102 |  | 0.058 | (-0.013, 0.129) | 0.108 |  | 393 |
| 25+ | 0.076 | 0.082 | 0.078 | 0.093 |  | 0.008 | (-0.045, 0.062) | 0.761 |  | 811 |
| Total | 0.074 | 0.071 | 0.074 | 0.096 |  | 0.025 | (-0.018, 0.068) | 0.259 |  | 1,204 |
| Red, swelling or pus around eyes |  |  |  |  |  |  |  |  |  |  |
| 15-24 | 0.053 | 0.038 | 0.038 | 0.081 |  | 0.057 | (-0.005, 0.120) | 0.070 |  | 393 |
| 25+ | 0.048 | 0.033 | 0.051 | 0.060 |  | 0.025 | (-0.017, 0.068) | 0.242 |  | 811 |
| Total | 0.050 | 0.034 | 0.047 | 0.066 |  | 0.036 | (-0.002, 0.073) | 0.065 |  | 1,204 |
| Redness, swelling, pus, bad smell  around belly button or cord |  |  |  |  |  |  |  |  |  |  |
| 15-24 | 0.028 | 0.006 | 0.017 | 0.024 |  | 0.029 | (-0.308, 0.365) | 0.866 |  | 393 |
| 25+ | 0.029 | 0.010 | 0.014 | 0.018 |  | 0.022 | (-0.003, 0.047) | 0.090 |  | 811 |
| Total | 0.027 | 0.009 | 0.014 | 0.019 |  | 0.023 | (-0.005, 0.051) | 0.104 |  | 1,204 |

Notes: Data pertain to male partners with live-born babies. The analysis is based on panel data and conducted with xtlogit and margins commands in Stata 17. Each model controls for the following baseline characteristics: single years of age, household wealth, male partner’s years of schooling, marital status, both parents with secondary/higher education, always lived in locality of residence, employed in the past 12 months, weekly exposure to television, first-time father, relationship satisfaction, and perceived power in the relationship.

Table S5. Impact estimate for disagreement with statements representing components of the GEM scale, male partners of first-time mothers, by age group, Kinshasa 2020

|  | **Adjusted Means** | | | | **Impact** | | |  |
| --- | --- | --- | --- | --- | --- | --- | --- | --- |
|  | **Before/Baseline** | | **After/Endline** | |  |  |  |  |
| **Component** | **Comparison** | **Intervention** | **Comparison** | **Intervention** | **ATE** | **95% CI** | **p-value** | **N** |
| A women's most important role  is to take care of the home and cook |  |  |  |  |  |  |  |  |
| 15-24 | 1.776 | 1.488 | 1.591 | 1.413 | 0.109 | (-0.117, 0.335) | 0.344 | 393 |
| 25+ | 1.759 | 1.471 | 1.637 | 1.447 | 0.098 | (-0.058, 0.254) | 0.218 | 811 |
| Total | 1.761 | 1.480 | 1.620 | 1.438 | 0.099 | (-0.029, 0.228) | 0.128 | 1,204 |
| Men need sex more than women do |  |  |  |  |  |  |  |  |
| 15-24 | 1.848 | 1.781 | 1.771 | 1.751 | 0.047 | (-0.177, 0.271) | 0.683 | 393 |
| 25+ | 1.881 | 1.940 | 1.803 | 1.906 | 0.044 | (-0.114, 0.202) | 0.589 | 811 |
| Total | 1.872 | 1.885 | 1.794 | 1.851 | 0.045 | (-0.084, 0.174) | 0.497 | 1,204 |
| You don't talk about sex, you just do it |  |  |  |  |  |  |  |  |
| 15-24 | 2.411 | 2.398 | 2.452 | 2.469 | 0.030 | (-0.197, 0.256) | 0.797 | 393 |
| 25+ | 2.519 | 2.405 | 2.489 | 2.567 | 0.192 | (0.040, 0.344) | 0.013 | 811 |
| Total | 2.485 | 2.403 | 2.477 | 2.534 | 0.139 | (0.013, 0.265) | 0.031 | 1,204 |
| There are times when a woman  deserves to be beaten |  |  |  |  |  |  |  |  |
| 15-24 | 2.115 | 2.054 | 2.212 | 2.170 | 0.019 | (-0.200, 0.237) | 0.867 | 393 |
| 25+ | 2.355 | 2.388 | 2.389 | 2.430 | 0.008 | (-0.130, 0.146) | 0.909 | 811 |
| Total | 2.279 | 2.274 | 2.333 | 2.342 | 0.014 | (-0.103, 0.131) | 0.815 | 1,204 |
| Changing diapers, giving a bath,  and feeding kids is the mother’s  responsibility |  |  |  |  |  |  |  |  |
| 15-24 | 1.693 | 1.383 | 1.704 | 1.711 | 0.318 | (0.094, 0.542) | 0.005 | 393 |
| 25+ | 1.744 | 1.559 | 1.795 | 1.761 | 0.152 | (-0.004, 0.308) | 0.057 | 811 |
| Total | 1.726 | 1.501 | 1.765 | 1.746 | 0.207 | (0.080, 0.335) | 0.001 | 1,204 |
| It is a woman’s responsibility to  avoid getting pregnant |  |  |  |  |  |  |  |  |
| 15-24 | 2.071 | 2.041 | 2.097 | 2.051 | -0.016 | (-0.257, 0.226) | 0.900 | 393 |
| 25+ | 2.147 | 2.083 | 2.214 | 2.213 | 0.064 | (-0.097, 0.224) | 0.436 | 811 |
| Total | 2.122 | 2.070 | 2.176 | 2.159 | 0.035 | (-0.099, 0.168) | 0.609 | 1,204 |
| A man should have the final  word about decisions in his home |  |  |  |  |  |  |  |  |
| 15-24 | 1.306 | 1.203 | 1.286 | 1.259 | 0.076 | (-0.078, 0.230) | 0.334 | 393 |
| 25+ | 1.400 | 1.370 | 1.375 | 1.364 | 0.020 | (-0.106, 0.146) | 0.755 | 811 |
| Total | 1.370 | 1.314 | 1.346 | 1.329 | 0.039 | (-0.059, 0.138) | 0.431 | 1,204 |
| Men are always ready to have sex |  |  |  |  |  |  |  |  |
| 15-24 | 1.736 | 1.825 | 1.690 | 1.881 | 0.102 | (-0.135, 0.338) | 0.399 | 393 |
| 25+ | 1.847 | 1.956 | 1.898 | 1.937 | -0.069 | (-0.228, 0.0890 | 0.392 | 811 |
| Total | 1.813 | 1.911 | 1.833 | 1.918 | -0.014 | (-0.145, 0.118) | 0.839 | 1,204 |
| A woman should tolerate violence  to keep her family together |  |  |  |  |  |  |  |  |
| 15-24 | 1.967 | 2.033 | 2.059 | 2.068 | -0.057 | (-0.290, 0.176) | 0.631 | 393 |
| 25+ | 2.066 | 2.160 | 2.206 | 2.288 | -0.013 | (-0.174, 0.149) | 0.879 | 811 |
| Total | 2.036 | 2.162 | 2.162 | 2.211 | -0.030 | (-0.162, 0.103) | 0.662 | 1,204 |
| If someone insults me, I will defend  my reputation, with force if I have to |  |  |  |  |  |  |  |  |
| 15-24 | 2.305 | 2.528 | 2.423 | 2.452 | -0.194 | (-0.419, 0.032) | 0.092 | 393 |
| 25+ | 2.404 | 2.463 | 2.443 | 2.500 | -0.002 | (-0.156, 0.152) | 0.981 | 811 |
| Total | 2.375 | 2.483 | 2.439 | 2.482 | -0.065 | (-0.192, 0.062) | 0.314 | 1,204 |
| To be a man, you need to be tough |  |  |  |  |  |  |  |  |
| 15-24 | 1.879 | 1.806 | 1.992 | 2.058 | 0.140 | (-0.112, 0.392) | 0.277 | 393 |
| 25+ | 2.073 | 1.883 | 2.066 | 2.160 | 0.283 | (0.119, 0.448) | 0.001 | 811 |
| Total | 2.013 | 1.857 | 2.043 | 2.125 | 0.238 | (0.099, 0.377) | 0.001 | 1,204 |

Notes: Data pertain to male partners with live-born babies. The higher is the adjusted mean, the more gender-equitable is the male partner’s attitude.

Each component is coded as follows: 1=Totally agree; 2=Partially agree; 3=Disagree. The analysis is based on panel data and is conducted with xtreg and margins commands in Stata 17. Each model controls for the following baseline characteristics: single years of age, household wealth, male partner’s years of schooling, marital status, both parents with secondary/higher education, always lived in locality of residence, employed in the past 12 months, weekly exposure to television, first-time father, and perceived power in the relationship.

Table S6. Average treatment effects (ATE) for individual components of male involvement and shared decision-making indices, male partners of first-time mothers, by age group, Kinshasa 2020

|  | **15-24** | | |  | **25+** | | |  | **Total** | | |
| --- | --- | --- | --- | --- | --- | --- | --- | --- | --- | --- | --- |
| **Component** | **ATE** | **95% CI** | **p-value** |  | **ATE** | **95% CI** | **p-value** |  | **ATE** | **95% CI** | **p-value** |
| **Male involvement in ANC** |  |  |  |  |  |  |  |  |  |  |  |
| Sit in the consultation room with  FTM during the check-up | 0.085 | (0.016, 0.155) | 0.016 |  | 0.083 | (0.029, 0.136) | 0.002 |  | 0.077 | (0.034, 0.120) | <0.001 |
| Listen to the fetal heartbeat | 0.095 | (0.033, 0.158) | 0.003 |  | 0.072 | (0.027, 0.118) | 0.002 |  | 0.073 | (0.036, 0.110) | <0.001 |
| HIV or STI testing | 0.078 | (0.010, 0.147) | 0.026 |  | 0.066 | (0.009, 0.122) | 0.022 |  | 0.065 | (0.021, 0.109) | 0.004 |
| Ask provider if baby is healthy | 0.172 | (0.096, 0.248) | <0.001 |  | 0.131 | (0.070, 0.192) | <0.001 |  | 0.138 | (0.090, 0.186) | <0.001 |
| Ask provider if the baby is a boy or  a girl | 0.115 | (0.047, 0.183) | 0.001 |  | 0.064 | (0.009, 0.118) | 0.022 |  | 0.076 | (0.034, 0.119) | <0.001 |
| Ask provider about health problems  during pregnancy | 0.069 | (0.001, 0.137) | 0.047 |  | 0.117 | (0.060. 0.174) | <0.001 |  | 0.096 | (0.052, 0.141) | <0.001 |
| Ask provider about sex during  pregnancy | 0.092 | (0.022, 0.163) | 0.010 |  | 0.096 | (0.041, 0.152) | 0.001 |  | 0.093 | (0.049, 0.136) | <0.001 |
| Ask provider about what FTM should  eat during pregnancy | 0.131 | (0.059, 0.202) | <0.001 |  | 0.104 | (0.049, 0.160) | <0.001 |  | 0.109 | (0.065, 0.153) | <0.001 |
|  | | | | | | | | | | | |
| **Male involvement in birth planning** | |  |  |  |  |  |  |  |  |  |  |
| Find information about the pregnancy | 0.089 | (-0.008, 0.186) | 0.072 |  | 0.009 | (-0.060, 0.077) | 0.806 |  | 0.039 | (-0.017, 0.095) | 0.174 |
| Make decisions about antenatal care | 0.115 | (0.016, 0.213) | 0.023 |  | 0.042 | (-0.025, 0.109) | 0.223 |  | 0.064 | (0.008, 0.120) | 0.025 |
| Make a birth plan | 0.057 | (-0.040, 0.155) | 0.248 |  | 0.044 | (-0.023, 0.111) | 0.194 |  | 0.056 | (-0.004, 0.107) | 0.068 |
| Save money for emergencies | 0.080 | (-0.015, 0.176) | 0.099 |  | 0.029 | (-0.033, 0.090) | 0.363 |  | 0.054 | (0.002, 0.107) | 0.043 |
| Arrange transport for delivery | 0.053 | (-0.047, 0.152) | 0.301 |  | 0.058 | (-0.011, 0.126) | 0.100 |  | 0.062 | (0.005, 0.118) | 0.033 |
| Decide on skilled attendance at delivery | 0.109 | (0.020, 0.197) | 0.016 |  | 0.153 | (0.091, 0.215) | <0.001 |  | 0.137 | (0.087, 0.188) | <0.001 |
|  | | | | | | | | | | | |
| **Male engagement in newborn care** | |  |  |  |  |  |  |  |  |  |  |
| Change the baby’s diapers | 0.122 | (0.022, 0.221) | 0.017 |  | 0.098 | (0.032, 0.164) | 0.004 |  | 0.103 | (0.048, 0.157) | <0.001 |
| Help/support feeding | 0.046 | (-0.042, 0.134) | 0.303 |  | 0.063 | (0.009, 0.117) | 0.021 |  | 0.054 | (0.007, 0.100) | 0.023 |
| Help when baby cries | 0.082 | (0.010, 0.154) | 0.025 |  | 0.065 | (0.023, 0.106) | 0.002 |  | 0.069 | (0.032, 0.105) | <0.001 |
| Bathe the baby | 0.130 | (0.031, 0.229) | 0.010 |  | 0.108 | (0.040, 0.176) | 0.002 |  | 0.112 | (0.056, 0.168) | <0.001 |
| Play with the baby | 0.068 | (-0.001, 0.137) | 0.052 |  | 0.022 | (-0.017, 0.060) | 0.268 |  | 0.035 | (0.002, 0.068) | 0.039 |
| Look after the baby when the FTM  goes out to or is at work | 0.053 | (-0.031, 0.138) | 0.214 |  | 0.048 | (-0.001, 0.096) | 0.054 |  | 0.047 | (0.004, 0.090) | 0.030 |
| Cook food | 0.006 | (-0.091, 0.102) | 0.906 |  | 0.081 | (0.012, 0.149) | 0.021 |  | 0.056 | (-0.001, 0.111) | 0.052 |
| Wash the baby's clothes | 0.063 | (-0.029, 0.155) | 0.180 |  | 0.069 | (0.000, 0.138) | 0.049 |  | 0.069 | (0.013, 0.125) | 0.015 |
| Put the baby to sleep | 0.072 | (-0.005, 0.149) | 0.067 |  | 0.007 | (-0.038, 0.052) | 0.767 |  | 0.027 | (-0.012, 0.066) | 0.181 |
| Stay home when the child is/was sick? | 0.041 | (-0.051, 0.132) | 0.381 |  | 0.108 | (0.053, 0.163( | <0.001 |  | 0.082 | (0.035, 0.130) | 0.001 |
| Take the baby to the doctor | 0.032 | (-0.044, 0.110) | 0.407 |  | 0.041 | (-0.003, 0.083) | 0.066 |  | 0.038 | (-0.000, 0.075) | 0.050 |
|  | | | | | | | | | | | |
| **MNH-related shared decision making** | |  |  |  |  |  |  |  |  |  |  |
| When to start seeking ANC | 0.037 | (-0.047, 0.121) | 0.390 |  | 0.024 | (-0.046, 0.094) | 0.502 |  | 0.023 | (-0.032, 0.077) | 0.417 |
| Number of ANC visits | 0.048 | (-0.028, 0.124) | 0.218 |  | 0.032 | (-0.035, 0.099) | 0.348 |  | 0.030 | (-0.020, 0.082) | 0.232 |
| Where to deliver the baby | 0.065 | (-0.006, 0.135) | 0.074 |  | 0.041 | (-0.023, 0.105) | 0.208 |  | 0.046 | (-0.003, 0.095) | 0.066 |
| How soon to start breastfeeding | 0.010 | (-0.053, 0.073) | 0.758 |  | -0.035 | (-0.098, 0.028) | 0.277 |  | -0.022 | (-0.070, 0.026) | 0.363 |
| Whether to practice exclusive  breastfeeding | -0.019 | (-0.102, 0.064) | 0.656 |  | -0.022 | (-0.088, 0.044) | 0.511 |  | -0.022 | (-0.074, 0.031) | 0.423 |
| Umbilical cord care | -0.008 | (-0.083, 0.067) | 0.838 |  | 0.003 | (-0.054, 0.059) | 0.926 |  | -0.002 | (-0.047, 0.043) | 0.924 |
| Care seeking for obstetric danger signs | 0.003 | (-0.092, 0.098) | 0.953 |  | 0.012 | (-0.065, 0.089) | 0.761 |  | 0.000 | (-0.060, 0.060) | 0.995 |
| Birth spacing | -0.055 | (-0.141, 0.031) | 0.210 |  | -0.013 | (-0.087, 0.062) | 0.736 |  | -0.032 | (-0.090, 0.025) | 0.267 |
| Care seeking for newborn danger signs | 0.022 | (-0.090, 0.135) | 0.697 |  | -0.009 | (-0.089, 0.071) | 0.832 |  | -0.006 | (-0.071, 0.059) | 0.854 |
|  |  |  |  |  |  |  |  |  |  |  |  |
| N | 393 | | |  | 811 | | |  | 1,204 | | |

Notes: Data pertain to male partners with live-born babies. ANC- antenatal care; FTM – first-time mother; MNH – maternal and neonatal health

The analysis is based on the endline survey data. All outcomes are binary. Each logistic treatment effects model uses inverse-probability weighting and controls for the following baseline characteristics: single years of age, household wealth, male partner’s years of schooling, marital status, both parents with secondary/higher education, residence with biological father while growing up, always lived in locality of residence, employed in the past 12 months, weekly exposure to television, first-time father, relationship satisfaction, high father involvement while growing up, and perceived power in the relationship.
